# Qualitative analysis of barriers and facilitators to healthcare engagement for people with injecting‐related invasive infections using a social ecological framework

**DOI:** 10.1111/add.70175

**Published:** 2025-08-15

**Authors:** Lucy O. Attwood, Sophia E. Schroeder, Olga Vujovic, Andrew J. Stewardson, Joseph S. Doyle, Paul Dietze, Peter Higgs, Samantha Colledge‐Frisby

**Affiliations:** ^1^ Department of Infectious Diseases, The Alfred Hospital and School of Translational Medicine Monash University Melbourne Victoria Australia; ^2^ Disease Elimination Program Burnet Institute Melbourne Victoria Australia; ^3^ Department of Public Health La Trobe University Melbourne Victoria Australia; ^4^ National Drug Research Institute Curtin University Melbourne Victoria Australia

**Keywords:** endocarditis, hospitalisation, injecting drug use, models of care, sepsis, social ecological model

## Abstract

**Background and aims:**

Injecting‐related bacterial infections are increasing in many countries. Systemic infections often require prolonged treatment. Evidence suggests that people who inject drugs who have invasive infections are less likely to complete antimicrobial treatment and have poorer outcomes than patients without a history of injecting drug use. We used a social ecological model to identify critical barriers and facilitators that impact healthcare service access for people who inject drugs with an invasive infection.

**Design:**

A qualitative study using semi‐structured interviews.

**Setting:**

Melbourne, Victoria, Australia in 2023.

**Participants:**

Twenty participants were recruited from SuperMIX, a longitudinal cohort of people who inject drugs.

**Measurements:**

Thematic analysis used inductive coding to chart themes onto the core domains of the social ecological model.

**Findings:**

Participant experiences informed five key themes. (1) Health literacy influenced how participants responded to the *physical and experiential embodiment of symptoms*. (2) The *intersection between drug use and marginalisation* created compounding barriers to care. (3) *Familial and social embeddedness* of participants could both enable or restrict their healthcare access. (4) The use of *patient‐centred care to respond to intersecting needs* directly contributed to healthcare engagement outcomes. Finally, (5) trust was a critical dimension that influenced participants' experiences of healthcare access. While its presence or absence was felt at intrapersonal and interpersonal levels, *cultivating or discouraging trust* had its roots at the societal and institutional level.

**Conclusions:**

Among people who inject drugs, facilitators and barriers to seeking healthcare for invasive infections appear to be influenced by factors at all levels of the social ecological model (intrapersonal, interpersonal, institutional and societal).

## INTRODUCTION

Hospitalisations for injecting‐related infections among people who inject drugs have increased in many countries over the past decade [1, 2, 3]. These infections vary in severity, from skin and soft tissue infections to invasive infections, such as infective endocarditis, bone and joint infections, and bloodstream infections, and can require prolonged treatment [1, 4].

Whilst the population of people who inject drugs in North America appears to be growing and is correlated with the ‘opioid epidemic’ and prevalent use of fentanyl [5, 6, 7], Australia currently has relatively little fentanyl and no evidence of increasing numbers of people who inject drugs [8, 9]. Australia also provides universal healthcare and a high level of harm reduction measures, such as needle and syringe programmes (NSPs) and access to opioid agonist treatment (OAT) [10]. Yet the incidence of hospital admissions related to invasive infections appears to be increasing in Australia [9]. It is likely that a combination of individual‐level and structural‐level factors is contributing to this increase.

Barriers to care for people who inject drugs are well documented and include stigma, inadequate pain relief, fear of withdrawal, system fragmentation, and healthcare availability and accessibility [11, 12, 13, 14, 15, 16, 17, 18, 19]. The risk of injecting‐related infections is also affected by social determinants of health, such as unstable housing, poverty and incarceration [20]. Unstable housing increases the risk of injecting in public, while incarceration limits access to sterile injecting equipment in the Australian context [14, 21]. Structural‐level factors can also influence access to care. For example, the criminalisation of drug use may deter caregivers (traditionally women) from seeking healthcare if there is fear of losing child custody [22]. Conversely, facilitators to healthcare engagement include the delivery of individualised, trauma‐informed care, prompt receipt of OAT and addiction medicine support, and the inclusion of peers [19, 23, 24, 25, 26, 27, 28]. However, despite growing recognition of invasive injecting‐related infections, there has been little exploration of the interplay between individual and system factors that contribute to accessing and remaining engaged in care.

This gap is particularly pressing considering the increasing incidence of such infections, which has generated calls for reform in both public health policy and healthcare delivery systems [29, 30, 31]. However, interventions still traditionally rely on individual behavioural changes [32, 33, 34, 35, 36, 37]. One way to consider the social determinants in the context of the health of people who inject drugs is through the social ecological model [38]. This model allows for examination of not only individual factors, but wider societal factors when considering the health seeking and engagement of people who inject drugs with injecting‐related infections [38]. It makes explicit the multiple levels that interconnect and influence health behaviours, from the individual to the structural [38, 39]. Assessing the barriers and facilitators of healthcare seeking through the lens of the social ecological model can allow the identification of key areas where public health research, intervention or policy design may be required [21, 40]. It also allows for identification of the relationships between the multiple factors that contribute to health behaviours [33].

The social ecological model describes four key levels when considering health behaviours: intrapersonal (individual), interpersonal (networks), institutional (organisations) and societal (social norms, policies and laws) [38, 39]. This model has been applied to other drug‐related harms, such as opioid misuse and overdose [33, 40, 41, 42], polysubstance use [43], engagement with opioid agonist therapy [44, 45, 46, 47, 48], crystal methamphetamine use for sex in men who have sex with men [49], prevention of HIV [50, 51] and treatment of hepatitis C [52]. A recent review of injecting‐related infections highlighted that to stem the tide of injecting‐related infections, changes are required to the social conditions within which people receive treatment for these infections [21]. However, despite the well documented poor health outcomes of people who inject drugs with invasive infections and the association of invasive infections with socioeconomic characteristics [53, 54, 55], there is little empirical research investigating the application of the social ecological model to injecting‐related infections. We therefore aimed to explore the experiences of people who inject drugs who sought and engaged in care for the management of invasive infections. We aimed to identify the barriers and facilitators to healthcare access, using the social ecological model to examine the multiple levels of influence and inform more effective, context‐specific interventions and policies.

## METHODS

### Theoretical framework

We utilised a social ecological model to explore the multiple levels that shape the barriers and facilitators of seeking healthcare from the perspective of people who inject drugs with an invasive infection. Data were examined as relevant to each of the four levels of the model – intrapersonal (e.g. an individual’s experience and understanding of their infection), interpersonal (e.g. interactions with healthcare workers), institutional (e.g. access to evidence‐based treatment in hospital) and societal (e.g. broader policies, including the criminalisation of drug use and social housing policy.) The social ecological model expects that factors at each of these levels influence one another [38], and our analysis sought to explore these inter‐relationships. While the social ecological model demonstrated here has similarities to those used to study opioid misuse [40] and the HIV epidemic [51], based on feedback from interview participants we have specifically focused the model to highlight factors that may influence a participant seeking or engaging in healthcare.

### Setting and participants

**FIGURE 1:**
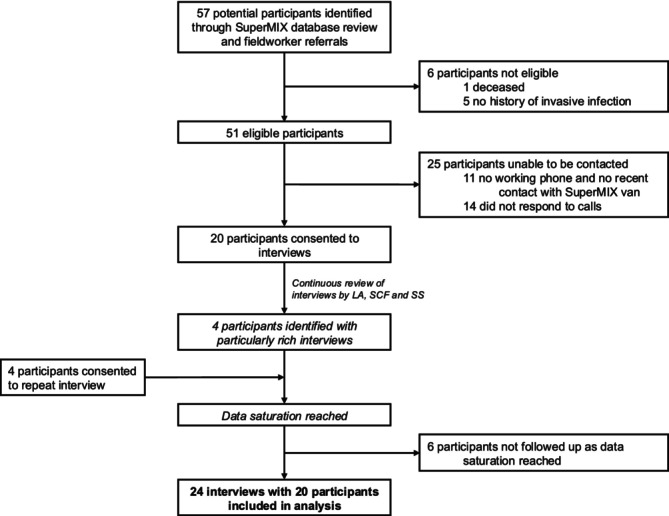
Participant screening and recruitment process.

Participants were purposively recruited from the SuperMIX cohort study, a large cohort of people who inject drugs recruited from 2008 onwards, and ongoing, in Melbourne, Victoria [56]. Recruitment occurred between April and August 2023. The key inclusion criteria were: aged at least 18 years; enrolled in the SuperMIX cohort study; and previous hospitalisation with an invasive infection related to injecting drug use. No criteria were set for time since hospitalisation as our research aim focused on the experience people recalled from their hospitalisation that may still be influencing their interactions with healthcare. When participants described multiple hospitalisations, we focused on their most recent experience. Potential participants were identified by interrogating the SuperMIX self‐report database for participants with a history of infective endocarditis and/or hospitalisation. Fieldworkers were also asked to notify study authors of eligible participants. A total of 57 potential participants were identified (Figure 1) and author S.S. made a minimum of three contact attempts to invite them to be part of the study. Participants who had had recent contact with fieldworkers were prioritised to facilitate more rapid recruitment and a concerted effort was made for an equal enrolment of genders. Recruitment ceased once L.A., S.S. and S.C.F. identified that data saturation relevant to the research questions had been reached. Authors L.A., S.S. and S.C.F. briefed potential participants on the study by telephone or in person and arranged a time for the interviews if they consented. S.C.F. and S.S. are postdoctoral researchers with extensive experience investigating injecting drug use behaviours and harms. L.A. is an infectious diseases physician completing a PhD on the management of invasive infections in people who inject drugs. These roles were explained to participants prior to consent being gained. Participants provided written or verbal informed consent prior to interviews commencing and received $AUD50 for their time and expertise.

Ethics approval for the study was granted by the Alfred Hospital Human Research Ethics Committee (Project 76/23).

### Data collection and management

In‐depth interviews were conducted by L.A., S.C.F. and S.S. using a semi‐structured interview guide (Table S1). The guide prompted interrogation into three main domains: injecting and clinical history before hospitalisation; experience accessing and receiving hospital care; and changes in health, health behaviours and drug use practices since hospitalisation. Questions were developed to allow fluid discussion of all levels of the social ecological model. We collected participant age, and the SuperMIX database was used to confirm age, sex and gender. The interview guide was used flexibly to allow participants’ experiences to be captured in their own words.

Four interviewees were invited to participate in a second interview as their original interviews contained particularly rich descriptions of their experiences and identified potential modifications to the healthcare system. In the follow‐up interviews the authors clarified key points in the interviewee narratives to gain a more detailed understanding of the participants’ experiences and their suggested methods to improve healthcare seeking. These participants were compensated a further $AUD50.

Participants were interviewed at locations convenient for them in metropolitan Melbourne or by telephone. The interviews lasted between 40 and 70 minutes. All interviews were audio‐recorded and transcribed verbatim by L.A., S.S. or S.C.F. All transcriptions were double‐checked by a second author to ensure accuracy. Identifiable information was removed from transcripts and a pseudonym was allocated to each participant. The non‐identifiable transcripts were uploaded into NVivoR1 for data processing and analysis.

### Analysis

L.A. led the analysis for this article, drawing on the thematic analysis approach developed by Braun and Clarke [57]. Interview transcripts were first subjected to an inductive analysis, as descriptive codes were assigned to the data ‘incident‐by‐incident’ [58] (Table S2). Codes were then collapsed further by comparing within and between individual participants’ narratives and considering the core concepts that unified related data segments. At each stage, authors L.A., S.S. and S.C.F. met regularly to review and refine codes and evolving themes. Subsequently, inductive codes were charted into the four theory‐informed domains (intrapersonal, interpersonal, institutional and societal factors), and themes were identified that captured the critical factors impacting the health behaviours of people experiencing injecting‐related invasive infections.

L.A. independently coded all data, whilst S.S. and S.C.F. independently coded interviews from 10 separate participants each for comparison, and critically reviewed the codes and early themes. Double coding was used to develop a coding framework that was then applied to all interviews. Any discrepancy in coding was discussed amongst L.A., S.S. and S.C.F. at regular meetings and consensus reached. The defining and naming of themes was led by L.A., as was the reporting of results. All authors discussed and agreed on the final themes presented in this article.

The authors ensured that the reporting of the study aligned with the Consolidated Criteria for Reporting Qualitative Studies (COREQ) qualitative checklist [59].

## RESULTS

We conducted 24 interviews with 20 participants. Table 1 describes the participant characteristics. Eleven men and nine women were interviewed, all of whom were cisgender, with a mean age of 43 years (IQR = 39–50 years). Ten participants (50%) had been hospitalised with infective endocarditis. Others were diagnosed with complicated skin and soft tissue infections (*n* = 3), bone and joint infections (*n* = 2), bloodstream infections (*n* = 2), complicated pneumonia (*n* = 2), and deep abscess (*n* = 1). A self‐reported diagnosis of pneumonia was considered an invasive infection when participants described treatment that was consistent either with pulmonary empyema or septic pulmonary emboli from infective endocarditis. Hospitalisation for an invasive infection within the past 12 months was reported by 12 participants (60%).

**TABLE 1:** Participant characteristics.

Pseudonym	Age (years)	Gender	Number of interviews	Self‐reported infection	Self‐reported length of time since hospitalisation for invasive infection	Self‐reported predominant substances injected
Cathy	33	F	1	Infective endocarditis	10 years	Heroin with Unisom^a^
Mike	46	M	1	Bone and joint infection	3 weeks	Heroin with Unisom, methamphetamines
Garry	49	M	1	Infective endocarditis	2 months	Heroin, methamphetamines
Veronica	44	F	1	Infective endocarditis	5 days	Heroin with Unisom, methamphetamines
Mila	52	F	1	Deep abscess	6 weeks	Heroin, methamphetamines
Nicole	42	F	2	Infective endocarditis	15 months	Heroin, methamphetamines
Russell	52	M	1	Infective endocarditis	12 years	Heroin, methamphetamines
Emma	40	F	1	Infective endocarditis	3 years	Heroin, methamphetamines
Alex	40	M	1	Pneumonia complicated by pulmonary embolism	18 months	Heroin, methamphetamines
Kate	42	F	2	Infective endocarditis	2 months	Heroin, methamphetamines
Louis	41	M	1	Infective endocarditis	6 years	Heroin with Unisom, methamphetamines
Alan	43	M	1	Complicated pneumonia	3 years	Heroin, methamphetamines
Laura	36	F	1	Infective endocarditis	6 months	Methamphetamines
Marcus	30	M	1	Complicated skin and soft tissue infection	6 weeks	Heroin with Unisom
Maeve	29	F	2	Complicated skin and soft tissue infection	4 weeks	Heroin with Unisom
Paul	53	M	1	Complicated skin and soft tissue infection	5 years	Heroin
Chris	57	M	1	Bone and joint infection	10 weeks	Heroin
Raymond	63	M	1	Bloodstream infection	2 years	Heroin
Rowan	46	M	2	Bloodstream infection	11 months	Heroin with Unisom
Alice	36	F	1	Infective endocarditis	8 years	Heroin with Unisom

^a^ Unisom is the brand name of the medication diphenhydramine, an antihistamine that can be purchased over the counter in Australia.

**FIGURE 2:**
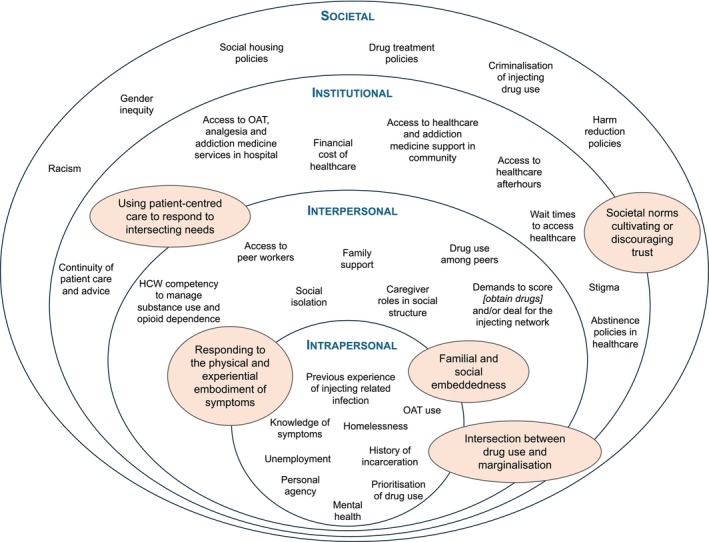
Social ecological model identifying factors contributing to healthcare utilisation in people who inject drugs and have invasive infections. Overarching themes are shaded in orange. Factors can be identified as a barrier, facilitator or both. Abbreviations: HCW, healthcare worker; OAT, opioid agonist therapy.

Analysis of the factors that influence a patient’s healthcare seeking identified five key themes. Figure 2 outlines how these themes map onto the social ecological model and identifies the factors that promote or limit participants’ healthcare engagement. Themes crossed over multiple levels of the social ecological model, highlighting the multidirectional links between factors that contribute to healthcare seeking and engagement for invasive infections. We describe these dynamics in detail below.

### Responding to the physical and experiential embodiment of symptoms

This theme identifies that the interpretation of symptoms by both patients and healthcare workers directly influences engagement in healthcare. Factors at the intra‐ and interpersonal level interact to form a framework by which patients and healthcare workers interpret and respond to symptoms. For most participants this framework was informed by their drug use, with gaps in health literacy regarding injecting‐related infections.

Many participants described having symptoms for many weeks to months before engaging in or accessing healthcare. Participants noted that they were unaware of the risk of invasive infections before experiencing their own, making the early interpretation of symptoms and signs difficult:

‘You don’t really hear much about endo[carditis] … until you’ve got it.’ (Russell, 52, infective endocarditis)

The interpretation of symptoms differed amongst participants. Symptoms of infection (e.g. sweating and fevers) can be confused as substance withdrawal. Thus, some participants denied having infective symptoms but instead described increasing their injecting frequency to address what they believed was withdrawal:

‘I did use a lot in the 48 hours before I went to hospital because I thought I was sick [withdrawing] for some reason. I didn’t think I had an infection …’ (Cathy, 33, infective endocarditis)

On the other hand, some participants detected their symptomology early and knew the next steps. These participants had either previous experience with infections or were acting on advice from trusted community healthcare workers. For example, one participant who had experienced multiple skin and soft tissue infections and was provided education on symptoms described now always seeking prompt treatment from her family doctor in the community:

‘[If] something’s gone real red and started to … be hot, then straight away I’ll go and get antibiotics and just take them. Cause yeah, it’s not fun to go to hospital for it.’ (Maeve, 29, complicated skin and soft tissue infection)

At the interpersonal level, participants’ narratives pointed to gaps in healthcare workers’ competency to manage substance use and withdrawal appropriately. As in‐patients, participants frequently described experiencing withdrawal and/or being denied analgesia (i.e. pain medication, such as morphine) through healthcare workers confusing OAT as analgesia:

‘To try to explain to some of the nurses that I have been on the same dose of methadone for how many years, it’s not a freaking pain relief … they didn’t get that.’ (Kate, 42, infective endocarditis)

For some participants, this not only hindered their ability to engage with healthcare workers, it also focused their priority to accessing drugs outside of the healthcare setting to prevent withdrawals rather than remaining engaged in care for their infection. For some, this resulted in early discharges and incomplete antimicrobial treatment.

### Intersection between drug use and marginalisation

A personal history of experiencing marginalisation through poverty and homelessness, intersecting with living experience of drug use, was described by participants as creating compounding barriers to care. Whilst this theme was not evident in all interviews, when it was present, it acted as a significant barrier to healthcare utilisation.

For some individuals, meeting the demands of their drug use in a context of competing psychosocial stressors, such as unstable housing, meant that their physical health (beyond any immediate withdrawal symptoms) was difficult to prioritise. The following quote identifies the daily struggle of surviving as a key reason for why symptoms went unattended:

‘I didn’t really notice any symptoms … I was homeless. I wasn’t eating … I was barely showering. I would have to occasionally pass the [convenience store] to think, “oh, I haven’t drank in a day. I need to eat.”’ (Kate, 42, infective endocarditis)

Kate went on to describe how these factors contributed to her illness and presentation:

‘I collapsed in somebody’s bathroom … they [the police] broke the bathroom door down and I was dead on arrival basically, so they had to resuscitate me.’

Kate’s presentation ultimately required admission to the intensive care unit, cardiac surgery and a 4‐month hospital admission.

Participants also experienced their marginalised social circumstances as a barrier to effective outpatient treatment. They predominantly described being managed as inpatients for the entirety of their therapy, with resultant prolonged admissions (up to 4 months in duration.) Only one participant was offered to continue antimicrobials through an outpatient parenteral antimicrobial therapy (OPAT) service. OPAT allows the delivery of intravenous antimicrobials as an outpatient at an infusion centre or, more typically in Australia, at a patients’ home [60]. However, social marginalisation often precluded involvement in these services. As explained by Garry (49, infective endocarditis):

‘That’s [OPAT] a possibility if you got stable accommodation, but technically when I went to hospital … I was homeless …’

When treatment delivery was coordinated with patients to account for individual circumstances, participants described fewer barriers to seeking or maintaining engagement. One participant described how a community health centre removed financial constrains to obtaining healthcare so that she could be reviewed by doctors:

‘I told them [the community health centre] I was coughing up blood … and so they actually paid for taxis for me to come back and see the doctors again. They knew I was really sick.’ (Nicole, 42, infective endocarditis)

Another participant who required 8 weeks of intravenous antimicrobials explained that his team facilitated the ability to leave the ward between antibiotic doses and organised the use of a peripherally inserted central catheter (PICC), which ‘made the antibiotic process much easier’ (Louis, 41, infective endocarditis). He completed the full course of intravenous antimicrobials in hospital.

### Familial and social embeddedness

Family and social relationships added a dynamic to participants’ experiences that could both enable or restrict their healthcare engagement. Several participants (predominantly female) identified that their families helped them remain engaged in care by offering perspective and providing practical or emotional support. One participant who described ‘… getting fed up with [not being able to go home] because I was like, “alright I’m going home this week” and then three weeks turn into four, five, six, seven, eight’ was able to identify that her mother had provided her critical support to enable her to remain engaged in care:

‘I was at the point of leaving and … my mum … convinced me to stay and said: “you have to stay there to get better for your daughter” … I knew I had to stay, I just really didn’t want to.’ (Cathy, 33, infective endocarditis)

However, the presence of family members impacted on participants’ ability to have open discussions with healthcare workers. Specifically, participants’ shame and fear of their family’s reactions prevented them from freely discussing their drug use. Cathy (33, infective endocarditis), also reflected that:

‘I think I was more truthful when my mum wasn’t there. Like I obviously wouldn’t have … told them everything while my mum was there.’

While family support typically enabled participants to seek or engage in treatment, most participants spoke of having minimal social support. Consequently, caring responsibilities precluded many, often mothers, from focusing on their health, as their priority was providing care for their family. As captured in the quote below, this single parent initiated an unplanned discharge 48 hours after her admission for infective endocarditis as there was no network of support that she could trust to look after her child while she was in hospital. She had not followed up with any care since:

‘Especially my daughter … I know, I’d be no good to her dead, but I’m no good to her being in hospital and her being out here on her own either. So, you’ve gotta put other things first before … it sounds silly, before your health.’ (Emma, 40, infective endocarditis)

Some hospitals facilitated assistance to socially isolated patients. Participants noted that the provision of support such as food vouchers and transportation enabled ongoing engagement in care. However, this assistance was only provided during inpatient or OPAT admissions. As one participant explained, even though he was expected to remain engaged in outpatient follow‐up, once OPAT was no longer required:

‘… all the help from the hospital, like home help, cleaning … all … that was all out the window. So when I got sick [with a further infection], I was, I was screwed. You know, I was here by myself, I had no help, nobody.’ (Chris, 57, bone and joint infection)

### Using patient‐centred care to respond to intersecting needs

This theme operates at the interpersonal and institutional level and describes how instances of good patient‐centred care, or lack thereof, contribute to healthcare engagement. A patient‐centred care approach involves clinicians treating not just a condition but valuing what is important to a patient as an individual. Its presence (or absence) was highlighted as a key facilitator (or barrier) to care by most participants.

Emergency departments (EDs) were frequently described as the first point of contact with healthcare for our participants. However, long wait times coupled with a lack of OAT created tension between the acute threat of withdrawal compared with the infectious symptoms participants had presented with. Ultimately, this led many to leave EDs before their assessments were completed:

‘… you can’t sit around [in the ED] forever. I think the first day that I was there, … I went in at like 3 o'clock in the afternoon. And it was 11 o'clock at night when I simply had to leave. I had to go and score [obtain drugs]. I was starting to hang out [experience withdrawal]. I was getting sicker [opioid withdrawal], and I was sick!’ (Garry, 49, infective endocarditis)

When participants’ healthcare experience included an appreciation of withdrawal risk and prompt review by specialised addictions teams, they described improved capacity and motivation to stay:

‘They [healthcare workers at a hospital] were just really good. And they made sure I had methadone so that I could stay, you know? … So that I could actually get … the whole course of the IV [intravenous] antibiotics … Which like, the other hospitals didn’t do.’ (Maeve, 29, complicated skin and soft tissue infection)

Participants often received care from multiple disparate teams during their admissions. They reported receiving conflicting advice from the various teams, which left them feeling overwhelmed and ultimately disengaged:

‘… there was probably ‘bout seven to eight different categories of doctors coming in. And giving me different information all the time. So I got to a point where I shut out … it was going through one ear and out the other.’ (Chris, 57, bone and joint infection)

Participants’ narratives demonstrated that navigating their follow‐up care was a complex and overwhelming experience. One participant described getting ‘bombarded with paperwork’ to attend a multitude of medical appointments in disparate locations post‐discharge. Without an adequate explanation of their purpose or scheduling, Laura (36, infective endocarditis) described a sense that:

‘… I was drowning … I need help from a nurse … to tell me what they [the investigations] are, so I know what I’m in for that day. And to help me work out the dates and times on paper, ‘cause I can’t have it like this. Nup – I can’t follow this … I’ll end up just throwing it all in the garbage bin.’ (Laura, 36, infective endocarditis)

Conversely, patient‐centred discharge plans that allowed follow‐up at services that were known to participants enabled ongoing engagement. As described by Nicole, cardiology review at her community health centre permitted continuity of care:

‘I get all my methadone and all that sort of stuff through [the community health centre] … my doctor, reached out for me to have an echo [echocardiogram], and then I … got a call to say I had an appointment for my heart again. So, everything that I have now is also interlinked.’ (Nicole, 42, infective endocarditis)

### Societal norms cultivating or discouraging trust

Trust was a critical dimension that influenced participants’ experiences, and this was evident in discussions with all participants. While its presence or absence was felt at intrapersonal and interpersonal levels, it was rooted in societal and institutional levels.

Participants often described not being believed when discussing the presence or severity of symptoms. Participants felt being misperceived as seeking analgesia when they were presenting unwell with infections. Some participants believed that their care was incomplete or neglected initially, and that this led to further medical complications. For example, Nicole (42, infective endocarditis) described receiving ‘incomplete blood testing’, owing to a lack of venous access. She subsequently suffered a stroke (a complication of infective endocarditis), which prompted her third presentation (and eventual admission) to hospital:

‘I ended up having a stroke. My daughter came in one morning and went to speak to me and apparently, I just wasn’t making any sense. And so the ambulance came for the third time. They kicked me out of hospital twice, thinking I was just seeking pain killers.’ (Nicole, 42, infective endocarditis)

Participants recalled experiencing a level of hostility directed towards them when navigating the healthcare environment. They perceived that they were considered responsible for potential antimicrobial failures and surgical complications owing to their drug use histories:

‘The other doctor [surgeon] really didn’t care. He was just like “whatever, this guy is a drug user and has got an infection on his heart” … he said something really terrible. I am sure it was probably about the most unprofessional thing I have heard of any doctor: “He will probably be dead in six months anyway.”’ (Garry, 49, infective endocarditis)

Another participant described a level of conditionality attributed to the surgical care offered, as the surgeon put forward an ultimatum between their drug use and healthcare:

‘He [the surgeon] sort of, … put it to me, … if I’m gonna keep using, he’s not gonna waste his time.’ (Chris, 57, bone and joint infection)

Overwhelmingly, participants felt an unmet need for someone to relate to their experience and advocate on their behalf. Participants felt that hospital peer navigators would more implicitly understand a patient’s situation and could help engagement by increasing patient trust:

‘I think it’s easier to take advice from someone who’s been there …’ (Maeve, 29, complicated skin and soft tissue infection)

Relatedly, it was noted that peers could also improve agency for participants because ‘then they could be the voice of the sick people’ (Kate, 42, infective endocarditis).

## DISCUSSION

Our analysis of the experiences of people who inject drugs hospitalised with invasive infections identified five themes that detail the multiple inter‐relationships present between all levels of the social ecological model (Figure 2). Facilitators to healthcare engagement include health and substance use literacy as well as individualised, patient‐centred care. Conversely, barriers such as distrust between patients and healthcare workers, marginalisation and social isolation often resulted in incomplete treatment or follow‐up. Below, we explore the facilitators and barriers to healthcare stratified by the social ecological model and demonstrate the utility of the model by linking potential interventions for responding to barriers and facilitators at each level of the model.

### Intrapersonal

Participants expressed that their understanding of symptoms directly facilitated or delayed their care seeking. Improving individuals’ knowledge of invasive infections is thus essential to promote early intervention [61, 62]. Education regarding sterile drug preparation, skin cleaning, safer drug injecting techniques and injecting‐related infections is required [35, 36, 37, 63]. However, research on these interventions demonstrates mixed results [35, 36, 37]. For long‐term impact, these interventions must also be coupled with the interpersonal, institutional and societal changes described below.

### Interpersonal

Our participants felt that healthcare workers failed to appreciate the complexities of their infectious symptoms, analgesia requirements and risk of opioid withdrawal. This is consistent with previous work [15, 64] and reflects the limited evidence‐based training most hospital staff receive on managing substance use disorders [65]. To mitigate this, changes at all levels of training are required to improve clinician understanding of substance use and the associated comorbidities experienced by these patients [30]. Training must also be supported by institutional guidelines on how to care for these patients, which is discussed below.

Social isolation, coupled with a perceived hostile, fragmented healthcare system, often meant participants reported feeling disempowered and unable to advocate for themselves. The inclusion of peer navigators has been demonstrated to assist with patient advocacy and patient–staff communication [23, 24], and was highlighted by Maeve and Kate above as a potential intervention. However, further research is required on how best to implement peer navigators in hospital settings to ensure that they are not at risk of stigma and burnout themselves [23].

### Institutional

Traditionally, the provision of hospital care to patients who use drugs is contingent on patient abstinence. Perceived deviations to abstinence are frequently met with punitive measures, such as threats to discharge, increased surveillance, behavioural contracts and visitor restriction [66, 67, 68]. Our study found abstinence‐based policies contributed directly to patient‐directed discharge, mistrust in the healthcare system and delayed care (e.g. in the case of Garry, described above). This substantiates previous research findings, which also identified that such policies exacerbated the confusion healthcare workers experienced regarding how, and if, they can provide harm reduction to patients [65, 67]. Institutions should invest in the development and implementation of hospital policies that are trauma‐informed, evidence‐based and uphold medical care as a fundamental human right, independent of a patient’s ability to abstain.

As highlighted by participants such as Maeve, the prompt receipt of OAT improved retention in healthcare. However, many participants described only being linked with addiction medicine services once admitted to wards. Our findings suggest addiction and harm reduction services should be available upon arrival to hospital. Addiction medicine services have been demonstrated to improve trust in healthcare workers [28], increase OAT uptake and increase antimicrobial completion for patients with invasive infections [69, 70]. The risk of additional injecting‐related harms could be further reduced by providing sterile supplies in hospital and/or access to safe injecting facilities [71]. Whilst we are aware of hospitals in Australia providing injecting equipment to patients on discharge, this is usually at the discretion of ward unit managers and is not supported by hospital policy. This reflects similar findings from North America, which demonstrate variability in supplying injecting equipment [72, 73]. In parts of Canada, NSPs and even supervised consumption services have been implemented at some hospitals for in‐patients [71, 74, 75].

Outpatient parenteral antimicrobial therapy (OPAT) is a patient‐centred model that can facilitate the receipt of intravenous antimicrobials outside of hospital. In Australia, this includes treatment at a specialised clinic or at a patient’s home [60, 76]. However, people who inject drugs are often excluded from OPAT services owing to concerns about ongoing substance use, staff safety and unstable housing [76, 77]. Discharge from inpatient hospital wards onto an OPAT service should not be categorically denied because of injecting drug use [62, 77], yet only one of our participants described being managed on an OPAT service. Multiple studies have demonstrated that people who inject drugs can complete therapy through OPAT, especially when support is provided [77, 78, 79]. Local investment and policies guiding the inclusion of people who inject drugs onto OPAT may not only improve patient retention in care but could also result in healthcare savings for institutions [77].

Alternatively, providing healthcare in low‐threshold environments, such as NSPs, could minimise the barriers to early care for injecting‐related infections [80]. NSPs may be able to offer treatment for simple infections such as skin and soft tissue infections, mediate access to tertiary healthcare services, when required, and assist with transition care post‐discharge [30, 81, 82]. This is particularly important with the increase in xylazine‐associated wounds in North America and increased non‐viral infections associated with the co‐injection of diphenhydramine gel capsules seen in Australia [83, 84].

### Societal

As long as injecting drug use remains criminalised, the provision of healthcare to people who inject drugs will be compromised by ethical debates [85]. Developing evidence‐based guidelines is similarly compromised whilst individuals are asked to disclose illegal activities for epidemiological data collection [8, 9]. To address the injecting‐related harms described by our participants, government funding needs to prioritise harm reduction and prevention over enforcing laws to suppress individuals’ drug use [86]. Furthermore, funding and surveillance activities need to broaden their focus from HIV and hepatitis C to include other injecting‐related infections [30, 87]. Research should also be responsive to emerging substances such as xylazine and nitazene opioids, where associated harms are less well understood [83].

Lack of appropriate housing is a key social determinant of the ill health experienced by our participants, whose healthcare seeking became secondary to meeting basic needs for food and shelter, as described by Kate. The UK saw a reduction in injecting‐related infections following the implementation of a social housing policy, with the provision of hotel‐based accommodation to the unstably housed, in response to COVID‐19 [88]. Considering the rising rates of homelessness in many nations [9, 14, 88], and the concurrent increase in injecting‐related infections, an urgent expansion of public housing programmes is required to address this key determinant of health [89, 90].

### Limitations

Participants in the SuperMIX cohort have agreed to participate in regular follow‐up surveys, and those more engaged in healthcare may have self‐selected. Participant recollection of events may be subject to recall bias or be impacted by more recent interactions with healthcare workers. We did not set a limit on the time since hospitalisation and thus participant recollections from more distant admissions may be more at risk of recall bias. It is possible that some participants may have an incomplete understanding of their infections and the treatment received. However, our primary aim was to understand participants’ perceptions regarding barriers and facilitators to care, and thus the exact diagnosis or treatment was not a key component of the analysis. Our cohort was predominantly white, and all spoke fluent English. Furthermore, all the interviewers are white females. People who inject drugs from different cultural backgrounds may experience different or additional barriers and facilitators to care not captured in our study. Potential bias in participant responses, including social desirability bias, could have occurred as we did not include a peer in our interview process, and one member of the research team conducting the interviews (L.A.) is a medical doctor. Furthermore, our personal understanding of invasive infections coupled with our research agenda may have contributed to interviewer bias. None of the authors conducting interviews are people with lived experience, and thus we can only discuss the experience of engaging with healthcare for invasive infections as outsiders.

## CONCLUSION

Our participants’ experiences demonstrated that healthcare engagement for invasive infections is impacted by interconnecting factors at every level of the social ecological model. This study demonstrated that the social ecological model provides a valuable framework through which the barriers and facilitators to seeking care for people who inject drugs and have invasive infections can be viewed. At an intrapersonal level, the health literacy of participants directly influenced their seeking healthcare. Both interpersonal and institutional level factors affected the delivery of patient‐centred care. Healthcare engagement was facilitated when care was provided with the recognition of a patient’s social circumstance and risk of withdrawal from drug use. Conversely, social isolation and the need for patients to navigate a complex, abstinence‐led healthcare system hindered the ability of our participants to engage in healthcare. These factors intersected with wider societal factors, such as the criminalisation of drug use, to create an environment that was hostile to healthcare engagement for our participants. Public health policy needs to encompass wider interpersonal, institutional and societal interventions to address injecting‐related infections in people who inject drugs. Further research is required to evaluate how best to implement patient‐centred models of care to improve the management of injecting‐related invasive infections. This study adds to previous work conducted in North America and Europe by demonstrating that structural‐level interventions are required to improve health outcomes, even in countries with high harm reduction access and universal healthcare, such as Australia.

## AUTHOR CONTRIBUTIONS

**Lucy O. Attwood:** Conceptualization (equal); data curation (equal); formal analysis (lead); investigation (equal); methodology (equal); writing—original draft (lead). **Sophia E. Schroeder:** Data curation (equal); formal analysis (equal); investigation (equal); methodology (equal); validation (equal); writing—review and editing (equal). **Olga Vujovic:** Supervision (equal); writing—review and editing (equal). **Andrew J. Stewardson:** Supervision (lead); writing—review and editing (equal). **Joseph S. Doyle:** Resources (equal); writing—review and editing (equal). **Paul Dietze:** Methodology (equal); project administration (equal); resources (equal); supervision (equal); writing—review and editing (equal). **Peter Higgs:** Conceptualization (equal); formal analysis (equal); methodology (equal); supervision (equal); writing—review and editing (equal). **Samantha Colledge‐Frisby:** Conceptualization (equal); formal analysis (equal); funding acquisition (lead); investigation (equal); project administration (equal); writing—review and editing (equal).

## DECLARATION OF INTERESTS

P.H.’s institution has received investigator‐initiated funding from Gilead Sciences and AbbVie for work on hepatitis C unrelated to this article. J.S.D has received research funding to his institution from Gilead Sciences and AbbVie, and consultancies to his institution from AbbVie. All other authors have no competing interests to declare.

## Supporting information


**Table S1.** Interview guide.

**Table S2.** Coding process with examples

## Data Availability

The data that support the findings of this study are available from the corresponding author upon reasonable request.

## References

[1] McCarthy NL , Baggs J , See I , Reddy SC , Jernigan JA , Gokhale RH , et al. Bacterial Infections Associated With Substance Use Disorders, Large Cohort of United States Hospitals, 2012‐2017. Clin Infect Dis. 2020;71(7):e37–e44. 31907515 10.1093/cid/ciaa008 PMC7900878

[2] Gomes T , Kitchen SA , Tailor L , Men S , Murray R , Bayoumi AM , et al. Trends in Hospitalizations for Serious Infections Among People With Opioid Use Disorder in Ontario. Can J Addict Med. 2022;16(4):433–439. 10.1097/ADM.0000000000000928 34711742 PMC9365258

[3] Langham FJ , Curtis SJ , Tang MJ , Jomon B , Doyle JS , Vujovic O , et al. Acute injection‐related infections requiring hospitalisation among people who inject drugs: Clinical features, microbiology and management. Drug Alcohol Rev. 2022;41(7):1543–1553. 10.1111/dar.13525 36053863 PMC9804300

[4] Delgado V , Ajmone Marsan N , de Waha S , Bonaros N , Brida M , Burri H , et al. 2023 ESC Guidelines for the management of endocarditis. Eur Heart J. 2023;44(39):3948–4042. 10.1093/eurheartj/ehad193 37622656

[5] Coyle JR , Freeland M , Eckel ST , Hart AL . Trends in Morbidity, Mortality, and Cost of Hospitalizations Associated With Infectious Disease Sequelae of the Opioid Epidemic. J Infect Dis. 2020;222(Suppl 5):S451–S457. 10.1093/infdis/jiaa012 32877550

[6] Schwetz TA , Calder T , Rosenthal E , Kattakuzhy S , Fauci AS . Opioids and Infectious Diseases: A Converging Public Health Crisis. J Infect Dis. 2019;220(3):346–349. 10.1093/infdis/jiz133 30941402 PMC6941614

[7] Hrycko A , Mateu‐Gelabert P , Ciervo C , Linn‐Walton R , Eckhardt B . Severe bacterial infections in people who inject drugs: the role of injection‐related tissue damage. Harm Reduct J. 2022;19(1):41. 10.1186/s12954-022-00624-6 35501854 PMC9063270

[8] Larney S , Peacock A , Mathers BM , Hickman M , Degenhardt L . A systematic review of injecting‐related injury and disease among people who inject drugs. Drug Alcohol Depend. 2017;171:39–49. 10.1016/j.drugalcdep.2016.11.029 28013096

[9] Attwood LO , O'Keefe D , Higgs P , Vujovic O , Doyle JS , Stewardson AJ . Epidemiology of acute infections in people who inject drugs in Australia. Drug Alcohol Rev. 2024;43(1):304–314. 10.1111/dar.13772 37995135 PMC10952783

[10] Colledge‐Frisby S , Ottaviano S , Webb P , Grebely J , Wheeler A , Cunningham EB , et al. Global coverage of interventions to prevent and manage drug‐related harms among people who inject drugs: a systematic review. Lancet Glob Health. 2023;11(5):e673–e683. 10.1016/S2214-109X(23)00058-X 36996860 PMC12765503

[11] Miller‐Lloyd L , Landry J , Macmadu A , Allard I , Waxman M . Barriers to Healthcare for People Who Inject Drugs: A Survey at a Syringe Exchange Program. Subst Use Misuse. 2020;55(6):896–899. 31902293 10.1080/10826084.2019.1710207

[12] van Boekel LC , Brouwers EP , van Weeghel J , Garretsen HF . Stigma among health professionals towards patients with substance use disorders and its consequences for healthcare delivery: systematic review. Drug Alcohol Depend. 2013;131(1–2):23–35. 10.1016/j.drugalcdep.2013.02.018 23490450

[13] Paquette CE , Syvertsen JL , Pollini RA . Stigma at every turn: Health services experiences among people who inject drugs. Int J Drug Policy. 2018;57:104–110. 10.1016/j.drugpo.2018.04.004 29715589 PMC5994194

[14] Harris RE , Richardson J , Frasso R , Anderson ED . Experiences with skin and soft tissue infections among people who inject drugs in Philadelphia: A qualitative study. Drug Alcohol Depend. 2018;187:8–12. 10.1016/j.drugalcdep.2018.01.029 29626746

[15] Summers PJ , Hellman JL , MacLean MR , Rees VW , Wilkes MS . Negative experiences of pain and withdrawal create barriers to abscess care for people who inject heroin. A mixed methods analysis. Drug Alcohol Depend. 2018;190:200–208. 10.1016/j.drugalcdep.2018.06.010 30055424

[16] Chan Carusone S , Guta A , Robinson S , Tan DH , Cooper C , O'Leary B , et al. “Maybe if I stop the drugs, then maybe they'd care?”‐hospital care experiences of people who use drugs. Harm Reduct J. 2019;16(1):16. 10.1186/s12954-019-0285-7 30760261 PMC6373073

[17] McNeil R , Small W , Wood E , Kerr T . Hospitals as a 'risk environment': an ethno‐epidemiological study of voluntary and involuntary discharge from hospital against medical advice among people who inject drugs. Soc Sci Med. 2014;105:59–66. 10.1016/j.socscimed.2014.01.010 24508718 PMC3951660

[18] Neale J , Tompkins C , Sheard L . Barriers to accessing generic health and social care services: a qualitative study of injecting drug users. Health Soc Care Community. 2008;16(2):147–154. 10.1111/j.1365-2524.2007.00739.x 18290980

[19] Eckland A , Kohut M , Stoddard H , Burris D , Chessa F , Sikka MK , et al. “I know my body better than anyone else”: a qualitative study of perspectives of people with lived experience on antimicrobial treatment decisions for injection drug use‐associated infections. Ther Adv Infect Dis. 2023;10:20499361231197065. 37693858 10.1177/20499361231197065 PMC10492466

[20] Degenhardt L , Peacock A , Colledge S , Leung J , Grebely J , Vickerman P , et al. Global prevalence of injecting drug use and sociodemographic characteristics and prevalence of HIV, HBV, and HCV in people who inject drugs: a multistage systematic review. Lancet Glob Health. 2017;5(12):e1192–e1207. 10.1016/S2214-109X(17)30375-3 29074409 PMC5683738

[21] Brothers TD , Bonn M , Lewer D , Comeau E , Kim I , Webster D , et al. Social and structural determinants of injection drug use‐associated bacterial and fungal infections: A qualitative systematic review and thematic synthesis. Addiction. 2023;118(10):1853–1877. 10.1111/add.16257 37170877

[22] Collins AB , Boyd J , Cooper HLF , McNeil R . The intersectional risk environment of people who use drugs. Soc Sci Med. 2019;234:112384. 31254965 10.1016/j.socscimed.2019.112384 PMC6719791

[23] Collins D , Alla J , Nicolaidis C , Gregg J , Gullickson DJ , Patten A , et al. “If It Wasn't for Him, I Wouldn't Have Talked to Them”: Qualitative Study of Addiction Peer Mentorship in the Hospital. J Gen Intern Med. 2019;1–8. 10.1007/s11606-019-05311-0 31512181

[24] Lennox R , Lamarche L , O'Shea T . Peer support workers as a bridge: a qualitative study exploring the role of peer support workers in the care of people who use drugs during and after hospitalization. Harm Reduct J. 2021;18(1):19. 10.1186/s12954-021-00467-7 33593364 PMC7885412

[25] Mahon D . A Systematic Review of Trauma Informed Care in Substance Use Settings. Community Ment Health J. 2025;61(4):734–753. 10.1007/s10597-024-01395-z 39641885

[26] French R , McFadden R , Stewart R , Christian H , Compton P . “I Just Need Proper Treatment”: Being Hospitalized for Endocarditis among Individuals Who Inject Drugs Being Hospitalized for Endocarditis. J Gen Intern Med. 2023;38(11):2470–2477. 10.1007/s11606-023-08133-3 36941420 PMC10465454

[27] Bearnot B , Mitton JA . “You're Always Jumping Through Hoops”: Journey Mapping the Care Experiences of Individuals With Opioid Use Disorder‐associated Endocarditis. J Addict Med. 2020;14(6):494–501. 32142056 10.1097/ADM.0000000000000648 PMC7483139

[28] King C , Collins D , Patten A , Nicolaidis C , Englander H . Trust in Hospital Physicians Among Patients With Substance Use Disorder Referred to an Addiction Consult Service: A Mixed‐methods Study. J Addict Med. 2022;16(1):41–48. 10.1097/ADM.0000000000000819 33577229 PMC8349928

[29] Serota DP , Barocas JA , Springer SA . Infectious Complications of Addiction: A Call for a New Subspecialty Within Infectious Diseases. Clin Infect Dis. 2020;70(5):968–972. 10.1093/cid/ciz804 31420651 PMC7319263

[30] Springer SA , Barocas JA , Wurcel A , Nijhawan A , Thakarar K , Lynfield R , et al. Federal and State Action Needed to End the Infectious Complications of Illicit Drug Use in the United States: IDSA and HIVMA's Advocacy Agenda. J Infect Dis. 2020;222(Suppl 5):S230–S238. 10.1093/infdis/jiz673 32877568 PMC7467230

[31] Wurcel AG . Rise in Endocarditis‐Related Hospitalizations in Young People Who Use Opioids: A Call to Action. Clin Infect Dis. 2020;72(10):1782–1783. 10.1093/cid/ciaa376 32270858

[32] Harvey L , Boudreau J , Sliwinski SK , Strymish J , Gifford AL , Hyde J , et al. Six Moments of Infection Prevention in Injection Drug Use: An Educational Toolkit for Clinicians. Open Forum. Infect Dis. 2022;9(2):ofab631. 10.1093/ofid/ofab631 PMC8794071 35097153

[33] Brady BR , Taj EA , Cameron E , Yoder AM , De La Rosa JS . A Diagram of the Social‐Ecological Conditions of Opioid Misuse and Overdose. Int J Environ Res Public Health. 2023;20(20):6950. 10.3390/ijerph20206950 37887688 PMC10606085

[34] Dwyer R , Topp L , Maher L , Power R , Hellard M , Walsh N , et al. Prevalences and correlates of non‐viral injecting‐related injuries and diseases in a convenience sample of Australian injecting drug users. Drug Alcohol Depend. 2009;100(1–2):9–16. 10.1016/j.drugalcdep.2008.08.016 19013725

[35] Phillips KT , Stewart C , Anderson BJ , Liebschutz JM , Herman DS , Stein MD . A randomized controlled trial of a brief behavioral intervention to reduce skin and soft tissue infections among people who inject drugs. Drug Alcohol Depend. 2021;221:108646. 33677353 10.1016/j.drugalcdep.2021.108646 PMC8055301

[36] Stein MD , Phillips KT , Herman DS , Keosaian J , Stewart C , Anderson BJ , et al. Skin‐cleaning among hospitalized people who inject drugs: a randomized controlled trial. Addiction. 2021;116(5):1122–1130. 10.1111/add.15236 32830383

[37] Roux P , Donadille C , Magen C , Schatz E , Stranz R , Curado A , et al. Implementation and evaluation of an educational intervention for safer injection in people who inject drugs in Europe: a multi‐country mixed‐methods study. Int J Drug Policy. 2021;87:102992. 33096364 10.1016/j.drugpo.2020.102992

[38] McLeroy KR , Bibeau D , Steckler A , Glanz K . An ecological perspective on health promotion programs. Health Educ Q. 1988;15(4):351–377. 3068205 10.1177/109019818801500401

[39] Bronfenbrenner U . Toward an experimental ecology of human development. Am Psychol. 1977;32(7):513–531.

[40] Jalali MS , Botticelli M , Hwang RC , Koh HK , McHugh RK . The opioid crisis: a contextual, social‐ecological framework. Health Res Policy Syst. 2020;18(1):87. 10.1186/s12961-020-00596-8 32762700 PMC7409444

[41] Dasgupta N , Beletsky L , Ciccarone D . Opioid Crisis: No Easy Fix to Its Social and Economic Determinants. Am J Public Health. 2018;108(2):182–186. 10.2105/AJPH.2017.304187 29267060 PMC5846593

[42] Cowan E , Khan MR , Shastry S , Edelman EJ . Conceptualizing the effects of the COVID‐19 pandemic on people with opioid use disorder: an application of the social ecological model. Addict Sci Clin Pract. 2021;16(1):4. 33413619 10.1186/s13722-020-00210-w PMC7789072

[43] Delcher C , Harris DR , Anthony N , Stoops WW , Thompson K , Quesinberry D . Substance use disorders and social determinants of health from electronic medical records obtained during Kentucky's “triple wave”. Pharmacol Biochem Behav. 2022;221:173495. 36427682 10.1016/j.pbb.2022.173495 PMC10082996

[44] Maina G , Marshall K , Sherstobitof J . Untangling the Complexities of Substance Use Initiation and Recovery: Client Reflections on Opioid Use Prevention and Recovery From a Social‐Ecological Perspective. Subst Abus. 2021;15:11782218211050372. 10.1177/11782218211050372 PMC8524687 34675526

[45] Tran BX , Ohinmaa A , Mills S , Duong AT , Nguyen LT , Jacobs P , et al. Multilevel predictors of concurrent opioid use during methadone maintenance treatment among drug users with HIV/AIDS. PLoS ONE. 2012;7(12):e51569. 10.1371/journal.pone.0051569 23251580 PMC3520938

[46] Bunting AM , Oser CB , Staton M , Eddens KS , Knudsen H . Clinician identified barriers to treatment for individuals in Appalachia with opioid use disorder following release from prison: a social ecological approach. Addict Sci Clin Pract. 2018;13(1):23. 10.1186/s13722-018-0124-2 30509314 PMC6278109

[47] Stopka TJ , Estadt AT , Leichtling G , Schleicher JC , Mixson LS , Bresett J , et al. Barriers to opioid use disorder treatment among people who use drugs in the rural United States: A qualitative, multi‐site study. Soc Sci Med. 2024;346:116660. 38484417 10.1016/j.socscimed.2024.116660 PMC10997882

[48] Kahn LS , Wozniak ML , Doscher T , Moore C , Vest BM . Treatment Experiences Among People Who Use Opioids: A Social Ecological Approach. Qual Health Res. 2022;32(8–9):1386–1398. 10.1177/10497323221104315 35645402

[49] Treloar C , Hopwood M , Drysdale K , Lea T , Holt M , Dowsett GW , et al. Stigma as understood by key informants: A social ecological approach to gay and bisexual men's use of crystal methamphetamine for sex. Int J Drug Policy. 2021;94:103229. 33774423 10.1016/j.drugpo.2021.103229

[50] Rhodes T , Simic M . Transition and the HIV risk environment. BMJ. 2005;331(7510):220–223. 16037463 10.1136/bmj.331.7510.220 PMC1179776

[51] Baral S , Logie CH , Grosso A , Wirtz AL , Beyrer C . Modified social ecological model: a tool to guide the assessment of the risks and risk contexts of HIV epidemics. BMC Public Health. 2013;13:482. 23679953 10.1186/1471-2458-13-482 PMC3674938

[52] Harris M , Rhodes T . Hepatitis C treatment access and uptake for people who inject drugs: a review mapping the role of social factors. Harm Reduct J. 2013;10:7. 23651646 10.1186/1477-7517-10-7 PMC3686576

[53] Ganesh SS , Goldshear JL , Wilkins P , Kovalsky E , Simpson KA , Page CJ , et al. Risk Factors for Infective Endocarditis and Serious Injection Related Infections Among People Who Inject Drugs in Los Angeles, CA and Denver. CO Drug Alcohol Depend. 2025;269:112588. 39954415 10.1016/j.drugalcdep.2025.112588 PMC11955157

[54] Topp L , Iversen J , Conroy A , Salmon AM , Maher L , NSPs CoA . Prevalence and predictors of injecting‐related injury and disease among clients of Australia's needle and syringe programs. Aust N Z J Public Health. 2008;32(1):34–37. 10.1111/j.1753-6405.2008.00163.x 18290911

[55] Hotton A , Mackesy‐Amiti ME , Boodram B . Trends in homelessness and injection practices among young urban and suburban people who inject drugs: 1997‐2017. Drug Alcohol Depend. 2021;225:108797. 34102506 10.1016/j.drugalcdep.2021.108797 PMC9373853

[56] Van Den Boom W , Quiroga MDM , O'Keefe D , Kumar D , Hill PL , Scott N , et al. Cohort Profile: The Melbourne Injecting Drug User Cohort Study (SuperMIX). Int J Epidemiol. 2022;51(3):e123–e130. 10.1093/ije/dyab231 34961882

[57] Braun V , Clarke V . Using thematic analysis in psychology. Qual Res Psychol. 2006;3(2):77–101.

[58] Flick U . The SAGE Handbook of Qualitative Data Analysis London: SAGE Publications Ltd; 2013. p. 664.

[59] Tong A , Sainsbury P , Craig J . Consolidated criteria for reporting qualitative research (COREQ): a 32‐item checklist for interviews and focus groups. International J Qual Health Care. 2007;19(6):349–357. 10.1093/intqhc/mzm042 17872937

[60] Norris AH , Shrestha NK , Allison GM , Keller SC , Bhavan KP , Zurlo JJ , et al. 2018 Infectious Diseases Society of America Clinical Practice Guideline for the Management of Outpatient Parenteral Antimicrobial Therapy. Clin Infect Dis. 2019;68(1):e1–e35. 10.1093/cid/ciy745 30423035

[61] Chan CA , Minahan‐Rowley R , Biegacki ET , Sue KL , Weimer MB . Development of a Patient and Clinician Informed Website on Injection Drug Use Related Infective Endocarditis. Subst Use Addctn J. 2024;29767342241267077. 10.1177/29767342241267077 39087514

[62] Baddour LM , Weimer MB , Wurcel AG , McElhinney DB , Marks LR , Fanucchi LC , et al. Management of Infective Endocarditis in People Who Inject Drugs: A Scientific Statement From the American Heart Association. Circulation. 2022;101161CIR0000000000001090. 10.1161/CIR.0000000000001090 36043414

[63] Kesten J , Hussey D , Lord C , Roberts L , Bayliss J , Erswell H , et al. Development, acceptability and feasibility of a personalised, behavioural intervention to prevent bacterial skin and soft tissue infections among people who inject drugs: a mixed‐methods Person‐Based Approach study. Harm Reduct J. 2023;20(1):114. 10.1186/s12954-023-00823-9 37608267 PMC10463350

[64] Compton P , Aronowitz SV , Klusaritz H , Anderson E . Acute pain and self‐directed discharge among hospitalized patients with opioid‐related diagnoses: a cohort study. Harm Reduct J. 2021;18(1):131. 10.1186/s12954-021-00581-6 34915913 PMC8679978

[65] Hyshka E , Morris H , Anderson‐Baron J , Nixon L , Dong K , Salvalaggio G . Patient perspectives on a harm reduction‐oriented addiction medicine consultation team implemented in a large acute care hospital. Drug Alcohol Depend. 2019;204:107523. 31541875 10.1016/j.drugalcdep.2019.06.025

[66] Englander H , Thakrar AP , Bagley SM , Rolley T , Dong K , Hyshka E . Caring for Hospitalized Adults With Opioid Use Disorder in the Era of Fentanyl: A Review. JAMA Intern Med. 2024;184(6):691–701. 38683591 10.1001/jamainternmed.2023.7282

[67] Strike C , Robinson S , Guta A , Tan DH , O'Leary B , Cooper C , et al. Illicit drug use while admitted to hospital: Patient and health care provider perspectives. PLoS ONE. 2020;15(3):e0229713. 10.1371/journal.pone.0229713 32134973 PMC7058273

[68] Huxley‐Reicher Z , Puglisi LB , Tetrault JM , Weimer MB , Stellini M , Bhandary‐Alexander J , et al. Response to substance use during hospitalization: A survey study of current and ideal policies and practices. J Hosp Med. 2023;18(9):829–834. 37475186 10.1002/jhm.13162

[69] Lewis S , Liang SY , Schwarz ES , Liss DB , Winograd RP , Nolan NS , et al. Patients With Serious Injection Drug Use‐Related Infections who Experience Patient‐Directed Discharges on Oral Antibiotics Have High Rates of Antibiotic Adherence but Require Multidisciplinary Outpatient Support for Retention in Care. Open Forum. Infect Dis. 2022;9(2):ofab633. 10.1093/ofid/ofab633 PMC8801224 35106316

[70] Marks LR , Munigala S , Warren DK , Liang SY , Schwarz ES , Durkin MJ . Addiction Medicine Consultations Reduce Readmission Rates for Patients With Serious Infections From Opioid Use Disorder. Clin Infect Dis. 2019;68(11):1935–1937. 10.1093/cid/ciy924 30357363 PMC6522678

[71] Brooks HL , Speed KA , Dong K , Salvalaggio G , Pauly BB , Taylor M , et al. Perspectives of patients who inject drugs on a needle and syringe program at a large acute care hospital. PLoS ONE. 2024;19(2):e0297584. 38359010 10.1371/journal.pone.0297584 PMC10868849

[72] Brothers TD , Mosseler K , Kirkland S , Melanson P , Barrett L , Webster D . Unequal access to opioid agonist treatment and sterile injecting equipment among hospitalized patients with injection drug use‐associated infective endocarditis. PLoS ONE. 2022;17(1):e0263156. 35081174 10.1371/journal.pone.0263156 PMC8791472

[73] Perera R , Stephan L , Appa A , Giuliano R , Hoffman R , Lum P , et al. Meeting people where they are: implementing hospital‐based substance use harm reduction. Harm Reduct J. 2022;19(1):14. 10.1186/s12954-022-00594-9 35139877 PMC8826677

[74] Nolan S , Kelian S , Kerr T , Young S , Malmgren I , Ghafari C , et al. Harm reduction in the hospital: An overdose prevention site (OPS) at a Canadian hospital. Drug Alcohol Depend. 2022;239:109608. 36063622 10.1016/j.drugalcdep.2022.109608 PMC9970047

[75] Dong KA , Brouwer J , Johnston C , Hyshka E . Supervised consumption services for acute care hospital patients. CMAJ. 2020;192(18):E476–E479. 10.1503/cmaj.191365 32366467 PMC7207181

[76] Attwood LO , McKechnie M , Vujovic O , Higgs P , Lloyd‐Jones M , Doyle JS , et al. Review of management priorities for invasive infections in people who inject drugs: highlighting the need for patient‐centred multidisciplinary care. Med J Aust. 2022;217(2):102–109. 10.5694/mja2.51623 35754144 PMC9539935

[77] Suzuki J , Johnson J , Montgomery M , Hayden M , Price C . Outpatient Parenteral Antimicrobial Therapy Among People Who Inject Drugs: A Review of the Literature. Open Forum. Infect Dis. 2018;5(9):ofy194. 10.1093/ofid/ofy194 PMC6127783 30211247

[78] Ho J , Archuleta S , Sulaiman Z , Fisher D . Safe and successful treatment of intravenous drug users with a peripherally inserted central catheter in an outpatient parenteral antibiotic treatment service. J Antimicrob Chemother. 2010;65(12):2641–2644. 10.1093/jac/dkq355 20864497

[79] O'Callaghan K , Tapp S , Hajkowicz K , Legg A , McCarthy KL . Outcomes of patients with a history of injecting drug use and receipt of outpatient antimicrobial therapy. Eur J Clin Microbiol Infect Dis. 2019;38(3):575–580. 10.1007/s10096-018-03461-3 30680563

[80] McNeil R , Small W . 'Safer environment interventions': a qualitative synthesis of the experiences and perceptions of people who inject drugs. Soc Sci Med. 2014;106:151–158. 10.1016/j.socscimed.2014.01.051 24561777 PMC4133147

[81] Castillo M , Ginoza MEC , Bartholomew TS , Forrest DW , Greven C , Serota DP , et al. When is an abscess more than an abscess? Syringe services programs and the harm reduction safety‐net: a case report. Harm Reduct J. 2020;17(1):34. 10.1186/s12954-020-00381-4 32487084 PMC7268493

[82] Bartholomew TS , Tookes HE , Chueng TA , Bluthenthal RN , Wenger LD , Kral AH , et al. Availability of telehealth‐based services at syringe services programs under the COVID‐19 Public Health Emergency. Harm Reduct J. 2023;20(1):122. 10.1186/s12954-023-00861-3 37660029 PMC10475193

[83] Zagorski CM , Hosey RA , Moraff C , Ferguson A , Figgatt M , Aronowitz S , et al. Reducing the harms of xylazine: clinical approaches, research deficits, and public health context. Harm Reduct J. 2023;20(1):141. 10.1186/s12954-023-00879-7 37777769 PMC10544173

[84] Higgs P , Dwyer R , Duong D , Thach ML , Hellard M , Power R , et al. Heroin‐gel capsule cocktails and groin injecting practices among ethnic Vietnamese in Melbourne. Aust Int J Drug Pol. 2009;20(4):340–346. 10.1016/j.drugpo.2008.05.001 18835767

[85] Wurcel AG , Zubiago J , Reyes J , Smyth E , Balsara KR , Avila D , et al. Surgeons' Perspectives on Valve Surgery in People With Drug Use‐Associated Infective Endocarditis. Ann Thorac Surg. 2023;116(3):492–498. 10.1016/j.athoracsur.2021.12.068 35108502 PMC9339044

[86] Ritter A , Grealy M , Kelaita P , Kowalski M . The Australian 'drug bduget': Government drug policy expenditure 2021/22 Sydney, Australia: Drug Policy Monitoring Program, University of New South Wales; 2024.

[87] Colledge‐Frisby S , Jones N , Larney S , Peacock A , Lewer D , Brothers TD , et al. The impact of opioid agonist treatment on hospitalisations for injecting‐related diseases among an opioid dependent population: A retrospective data linkage study. Drug Alcohol Depend. 2022;236:109494. 35605532 10.1016/j.drugalcdep.2022.109494

[88] Lewer D , Brothers TD , Croxford S , Desai M , Emanuel E , Harris M , et al. Opioid injection‐associated bacterial infections in England, 2002–2021: A time series analysis of seasonal variation and the impact of coronavirus disease 2019. Clin Infect Dis. 2023;77(3):338–345. 10.1093/cid/ciad144 36916065 PMC10425189

[89] Wickramatilake S , Zur J , Mulvaney‐Day N , Klimo MC , Selmi E , Harwood H . How States Are Tackling the Opioid Crisis. Public Health Rep. 2017;132(2):171–179. 10.1177/0033354916688206 28152337 PMC5349480

[90] Moore D , Dietze P . Enabling environments and the reduction of drug‐related harm: re‐framing Australian policy and practice. Drug Alcohol Rev. 2005;24(3):275–284. 16096131 10.1080/09595230500170258

