# Supplementary material for: Qualitative analysis of barriers and facilitators to healthcare engagement for people with injecting‐related invasive infections using a social ecological framework
Source: Addiction. 2025 Aug 15;120(12):2476–88. doi: 10.1111/add.70175 (PMC12586791; doi:10.1111/add.70175)
Supplement: Supplementary file 1 — Table S1. Interview guide. [file ADD-120-2476-s002.docx]

**Supplementary Table 1 - Interview guide**

***Opening script:***

Thanks for speaking with me today. To remind you, this is just an opportunity for you to tell me a bit about your experience of receiving care for your injecting-related infection. Some of these questions may be sensitive, or may touch on things that are upsetting. If you want to stop, or skip any questions, please just let me know.

Can I confirm that you are OK to be involved?

Are you okay with me audio recording this interview?

I am just going to note down the date and time of our interview, before we get started.

| Date of interview |  |
| --- | --- |
| Time of interview commencement |  |
| Basic information | 1. How old are you? 2. When were you hospitalised with an injecting-related infection? |
| Aim 1: Understand people’s experience of being hospitalized with an injecting-related infection (i.e. the ‘index’ event) | |
| Can you describe your most recent hospitalisation of an injecting-related infection? | 1. What were your reflections on your hospitalisation? 2. What were the symptoms that you experienced before going into hospital? (e.g., pus, heat, pain, swelling, tenderness) 3. What motivated you to seek hospital care? 4. How long did you wait until you went into hospital? Why? 5. Who was with you when you went to hospital? 6. What were the challenges/barriers you had in accessing hospital care/other care when you needed it for this infection? 7. How long were you in the Emergency Department for? How were you treated while in ED? 8. What were you diagnosed with and how was it explained to you? 9. Tell me about how you were cared for in hospital? And by whom? 10. What was your perception of the different people taking care of you while you were in hospital? 11. How was your infection treated? [e.g., oral antibiotics or surgical intervention] 12. What treatment options were presented to you? Did you feel like you had a choice on which treatment option to take? 13. Did you have a PICC line while in hospital? How did you and your healthcare team feel about needing a PICC line? 14. How did your healthcare providers investigate the source of your infection? 15. How was your injecting drug use discussed by you or your healthcare provider(s)? 16. How long were you in hospital? Were you able to leave the hospital/ward when you requested to? 17. Did you want to stay in hospital? Why or why not? 18. What were challenges of your time in hospital? 19. What would you want healthcare providers to know about people going through the experience you went through? 20. Did you receive any support from friends or family while you were in hospital?   *For those receiving opioid agonist treatment:*   1. Did you get any pain relief medication? 2. Were there any barriers to you receiving your drug treatment while in hospital? 3. What were the best and worst things about being in hospital this time? |
| What was your experience leaving hospital? | 1. How were you discharged? Where did you go? Was there any support at home (on the street etc) for you when you left? 2. Can you tell me about any follow-up treatment required?    1. Was the follow-up treatment accessible to you? What ways did you find worked best to stick to the treatment? 3. What follow-up was required? How did you manage this? 4. Were you offered the option of continuing antibiotics through a drip at home? If so, would you have preferred that and why? What challenges would this method have presented?   *For those who left against medical advice:*   1. What were the main reasons for self-discharge? 2. What might have had you complete your treatment in the hospital? |
| What instructions/ guidance/ recommendations were offered to you by your healthcare providers to follow post-release? | 1. How were these recommendations communicated to you? |
| Aim 2: Understand people’s history with injecting-related infections | |
| Can you tell me a bit about your injecting behaviour immediately before the hospitalisation? | 1. How often and what were you injecting? 2. How did you prepare your drugs and what injecting equipment did you use? 3. Did you ever have any issues with injecting (e.g., finding a vein, skin infections)? 4. What do you think might have contributed to the development of your infection? 5. Are you engaged with any services? (e.g., NSP, MSIR, drug treatment) 6. How long had you been injecting for? 7. Where do you usually get injecting equipment from? Did you ever have any issues getting new fits when you needed them? 8. Have you had any other injecting-related complications? (e.g., abscess, cellulitis, vein collapse, blood clots) Why do you think you have had those issues? |
| Thinking about this hospitalisation again, what was your experience before getting to hospital? | 1. How long did you have symptoms for? 2. What did you think of these symptoms? What was your understanding of the symptoms of an infection before this point? 3. What kinds of things did you do to make the symptoms worse / better? 4. How did you decide who to talk to about this infection? 5. Can you tell me about any attempts to access primary health care? 6. What was the result of that attempt to access care? |
| Aim 3: Understand the impact of going to hospital for an injecting-related infection on subsequent behaviour | |
| How did you recover after your hospitalisation? | 1. Were there any barriers/challenges during your recovery? 2. Were there any other difficulties about your hospitalisation? (e.g., not being able to access services, procure money, caring for others, etc.) 3. Was there anything positive about your experience (either during or after hospitalisation)? 4. What could the hospital have done to make it easier for your to be followed up after being sick? 5. (If on OAT) What kind of support was given to you to remain on pharmacotherapy/ OAT/ buprenorphine after leaving hospital? |
| Did your injecting behaviour change after your hospitalisation? | 1. Were there injecting sites you could not inject into? 2. Did you seek out safer/different injecting practices? 3. Did you continue to use as you previously had? |
| Closing questions | 1. Have you had a recurring infection? 2. If you had a friend with an infection, what would you tell them? 3. Do you know of other people who have been to hospital with an injecting-related infection? How has their experience compared to your own? |
| Do you have any questions for me? | |
| Would you like to be referred to telephone or face-to-face counselling? | |
| Length of interview | |
