# Supplementary material for: Qualitative analysis of barriers and facilitators to healthcare engagement for people with injecting‐related invasive infections using a social ecological framework
Source: Addiction. 2025 Aug 15;120(12):2476–88. doi: 10.1111/add.70175 (PMC12586791; doi:10.1111/add.70175)
Supplement: Supplementary file 2 — Table S2. Coding process with examples [file ADD-120-2476-s001.docx]

**Supplementary Table 2 – Coding process with examples**

| **Excerpt** | **Initial codes applied** | **Notes on code** | **Factors and level in SEM linked to code** | **Final theme** |
| --- | --- | --- | --- | --- |
| *“Yeah, um, while, like, while the infection was starting I used a massive amount um because I kept waking up through the night and thinking “I don’t know what’s wrong with me.” And then I thought “Oh maybe I’ll just have a little bit more and it will put me back to sleep or I’ll feel better.” So I kept doing that, I think it was for about 2 days until yeah I got to the point, I obviously had none left and I kept saying to my mum you know, “Just give me $50 and I’ll be alright” and she was like “no, you don’t look OK, there’s something wrong with you, you need to get on the methadone.” So I did use a lot, um, in the 48 hours before I went to hospital because I thought I was sick for some reason. I didn’t think I had an infection or something else was wrong with me.” (Cathy, 33, infective endocarditis)* | Health literacy; demands of drug use; late presentation; medical; addressing symptoms; injecting; duration of symptoms; support from family or friends; family; pharmacotherapy | Barrier - drug use impacting symptom interpretation, treated as ‘hanging out’  Facilitator – support from family (though still informed by drug use rather than knowledge of infection) | Intrapersonal - knowledge of symptoms; prioritisation of drug use; OAT use; personal agency  Interpersonal - family support | ***Responding to the physical and experiential embodiment of symptoms*** |
| *SS: “Hm. Um, so how did they prepare you for being discharged from hospital? What were some of the discussions you had?*  *R: So they gave me access to a social worker to talk about options for housing, um, ‘cause I was technically homeless.*  *P: A nurse in the home, did they come visit you?*  *R: Um, that’s a possibility if you got stable accommodation but technically when I went to hospital when I was admitted in [hospital] I was homeless. And had been for pretty much the whole of the year beforehand as well. Um, I had a period during that year where I was in stable accommodation but, um, yeah, I found out a couple of things about friends and getting robbed that year of which I wished I hadn’t had known.” (Garry, 49, infective endocarditis)* | Accommodation; discharge; support from HCW; isolation; discharge to OPAT; lack of choice; HCW not appreciating complexities of social situation; barriers to treatment in community | Barrier – unstable accommodation barrier to providing treatment through OPAT; long-standing pre-existing social stressors | Intrapersonal – homelessness  Interpersonal – drug use among peers; social isolation  Institutional – access to healthcare and addiction medicine support in community; continuity of patient care and advice  Societal – social housing policies | ***Intersection between drug use and marginalisation*** |
| *LA: “And so, with that experience in mind, if you had those symptoms again, would you be happy to go back to that hospital?*  *R: [Shakes head]*  *LA: No? Why’s that?*  *R: Now I just don't have time.*  *LA: Don’t have time?*  *R: Yeah.*  *LA: Cause of other things happening in your life?*  *R: Yeah. Especially my daughter. She’s got so much going on. You know, I know, I’d be no good to her dead, but I’m no good to her being in hospital and her being out here on her own either. So I’ve gotta put other things first before…It sounds silly, before your health. But when it comes to your kids and stuff, you’ll do anything. And I think I was just scared more than anything, I was just scared.” (Emma, 40, infective endocarditis)* | Care giving roles; family; feeling worried or scared; limited support; fear breads healthcare avoidance; complexities of social situation; impact of hospitalisation on healthcare seeking; late presentation; deflection or denial; social demands out of hospital; being scared to go to hospital; children as motivation; isolation | Barrier – lack of social supports to provide care for children when own health needs require hospitalisation | Intrapersonal – knowledge of symptoms; previous experience of injecting related infection  Interpersonal – caregiver roles in social structure; social isolation | ***Familial and social embeddedness*** |
| *LA: “…so now you've had that echo and it shows your valves leaking more than it should be now. Have they said what your next steps are now?*  *R: No. You, my doctor just said that we've just gotta sit and watch it, but um, he said that, if I have any worrying things like, you know, I'm getting pains in the chest or anything that even slightly, you know, uh, feels or looks like what it did when I had my last infection and things like that, um, just to call the ambulance straight away. Don't come to the doctor, just go straight, call an ambulance. But he said that, you know, it's nothing to worry about, like right now, it's not like, Oh my gosh. Straight away, another surgery right now. But he was just a bit blown away with how quick it has, yeah, it is running out.*  *LA: Yeah. And was that your heart doctor or your local doctor?*  *R: No, that's my heart doctor. But I see him at my local doctor's clinic.*  *LA: Ohh. OK. So he's not someone at the [hospital]. He's someone that you see at your local clinic?*  *R: Yeah, but he also works at the [hospital], but he comes just to my clinic in [suburb], like once every ...once a month I think?*  *LA: OK.*  *R: And then yeah, so.*  *LA: Yeah. And was that organised through the [hospital] or how did you get linked up with that?*  *R: That was through my doctor. I'm not sure which one. So I, my general, my local GP is through [community health centre] on [street]. And I get all my methadone and all that sort of stuff through [community health centre]. So it was one, over a year ago, so I think it was [name], my doctor, reached out for me to have an echo. And then, yeah, and then I just got a call to say I had an appointment for my heart again. So, everything that I have now is also interlinked. So everybody gets all the information through the doctors, the other doctors and through the hospitals and stuff. So I don't have to do much, they all just kind of intermingle with themselves.” (Nicole, 42, infective endocarditis)* | Ongoing follow up; logistical; health literacy; support from HCW; understanding of symptoms; surgery; facilitators; different teams in care; treatment in community | Facilitator – meeting patients where they are, providing care to trusted sites in community, educating patients on symptoms to be aware of | Interpersonal – HCW competency to manage substance use and opioid dependence  Institutional – Continuity of patient care and advice; financial costs of healthcare; access to healthcare and addiction support in community; wait times to access healthcare | ***Using patient-centred care to respond to intersecting needs*** |
| *“Look, the doctors at [hospital] are I would say incredibly professional. They actually care about people. Like, one female doctor, after the findings of my exploratory surgery or whatever session, where they pointed a camera in my heart, the next day... it was taken on a Friday, so I had to wait for my results on the Monday – thanks weekend! Yeah on that Monday a female Asian doctor, of Indian descent, she was really nice. She was really interested in how I was feeling and how I was going to deal with things when I was discharged. The other doctor really didn’t care. He was just like “whatever, this guy is a drug user and has got an infection on his heart.” Uh, I believe that he said something really terrible. I am sure it was probably about the most unprofessional thing I have heard of any doctor: “He will probably be dead in 6 months anyway.”” (Garry, 49, infective endocarditis)* | Delay to treatment from healthcare side; stigma; distrust; communication with HCW; medical; surgery; discharge planning; support from HCW; feeling trust in care provided; feeling HCW ‘actually care’; being treated ‘like a junkie’; being made responsible for potential treatment failure; feeling personally blamed for illness; positive experience in hospital | Facilitator – genuine care in patient and whole social situation shown by HCW; interest in how would manage post discharge  Barrier – assumptions made by HCW to patients outcome due to drug use; bias informing healthcare decisions | Institutional – stigma; abstinence policies in healthcare; access to healthcare and addiction medicine support in community  Societal – criminalisation of injecting drug use, drug treatment policies | ***Societal norms cultivating or discouraging trust*** |
